# Supplementary material for: DNA Methylation Patterns in Relation to Acute Severity and Duration of Anxiety and Depression
Source: Curr Issues Mol Biol. 2023 Sep 6;45(9):7286–303. doi: 10.3390/cimb45090461 (PMC10527760; doi:10.3390/cimb45090461)
Supplement: Supplementary file 1 [file cimb-45-00461-s001.zip › cimb-2569520-supplementary.pdf]

current issues in molecular biology

MDPI

Supplementary Materials for Article

DNA Methylation Patterns in Relation to Acute Severity and Duration of Anxiety and Depression

Eva Vidovič [1], Sebastian Pelikan [1], Marija Atanasova [2], Katarina Kouter [3,4], Indre Pileckyte [5], Aleš Oblak [1], Brigita Novak Šarotar [1,6], Alja Videtič Paska [3,*] and Jurij Bon [1,6]

1 University Psychiatric Clinic Ljubljana; Ljubljana, Slovenia
2 Faculty of Chemistry and Chemical Technology, University of Ljubljana; Ljubljana, Slovenia
3 Institute for Biochemistry and Molecular Genetics, Faculty of Medicine, University of Ljubljana; Ljubljana, Slovenia
4 Institute of Microbiology and Immunology, Faculty of Medicine, University of Ljubljana; Ljubljana, Slovenia
5 Center for Brain and Cognition, Pompeu Fabra University; Barcelona, Spain
6 Department of Psychiatry, Faculty of Medicine, University of Ljubljana; Ljubljana, Slovenia
* Correspondence: alja.videtic@mf.uni-lj.si

Abstract: Depression and anxiety are common mental disorders that often occur together. Stress is an important risk factor for both disorders, affecting pathophysiological processes through epigenetic changes that mediate gene-environment interactions. In this study, we explore two proposed models about the dynamic nature of DNA methylation (DNA methylation) in anxiety and depression: i.) a stable change in which DNA methylation accumulates over time as a function of the duration of clinical symptoms of anxiety and depression, and ii.) a flexible change in which DNA methylation correlates with the acute severity of clinical symptoms. Symptom severity was assessed using clinical questionnaires for anxiety and depression (BDI-II, IDS-C, HAM-A), and total lifetime symptom duration was obtained from patients' medical records. Peripheral blood DNA methylation levels were determined for the *BDNF*, *COMT*, and *SLC6A4* genes. We found a significant negative correlation between *COMT_1* amplicon methylation and acute symptom scores, with BDI-II, IDS-C, and HAM-A showing a similar degree of correlation. Our results suggest that DNA methylation follows flexible dynamics, with methylation levels closely associated with acute clinical presentation rather than duration of anxiety and depression. These results provide important insights into the dynamic nature of DNA methylation in anxiety and affective disorders and contribute to our understanding of the complex interplay between stress, epigenetics, and individual phenotype.

Keywords: Anxiety, depression, epigenetics, DNA methylation, *BDNF*, *COMT*, *SLC6A4*, symptom severity, symptom duration

Table S1. Demographic and clinical characteristics of participants.

ID	Gender	Age (Years)	IDS-C	BDI	HAM-A	CGI	Last Episode Duration (Months)	Lifetime Duration (Months)
s01	F	46	29	17	16	4	6	149
s02	F	67	40	45	24	4	6	6
s03	F	55	29	15	19	5	7	166
s04	F	60	61	46	33	7	3	84
s05	F	59	32	27	29	5	7	264
s06	F	42	25	14	11	4	3	167
s07	M	63	58	45	41	7	8	8
s08	F	58	27	19	7	4	5	236
s09	M	63	56	20	20	5	11	31
s10	M	79	25	22	10	5	3	120
s11	F	63	29	9	14	5	12	72
s12	F	47	54	35	45	5	10	21
s13	M	51	20	10	7	4	1	293
s14	M	28	31	46	21	6	7	31
s15	F	54	42	38	16	6	6	41
s16	F	51	47	30	25	5	24	33
s17	F	42	16	7	8	4	5	72
s18	F	55	30	39	24	4	2	175
s19	M	53	17	12	9	4	3	84
s20	M	37	10	9	2	4	5	216
s21	F	45	16	4	20	3	3	204

s22	F	45	31	17	20	4	6	84
s23	M	46	28	14	25	4	12	56
s24	F	63	26	17	22	4	9	214
s25	F	31	44	30	30	4	8	60

Table S2: Primer sequences (highlighted), NGS adapter (bold), annealing temperature, and amplicon length without adapters. N designates any base.

Amplicon	Forward (F) or Reverse (R) Primer	Primer Sequence	Annealing Temperature (°C)	Amplicon Length without Adapters (bp)
BDNF_1 [a]	F	TCGTCGGCAGCGTCAGATGTGTATAAGAGACAGTGAGGG-TAGGTAAAGGGTAGT	55.3	346
	R	GTCTCGTGGGCTCGGAGATGTGTATAAGAGA-CAACTCTCCCAAAAAACCCTAC		
BDNF_2 [a]	F	TCGTCGGCAGCGTCAGATGTGTATAAGAGACAGTTATA-TAGGTTTTTGTGGGTAATTAG	55	259
	R	GTCTCGTGGGCTCGGAGATGTGTATAAGAGA-CAGAAAAAAAAAAAACTTCTTAAAAAAAT		
BDNF_3 [a]	F	TCGTCGGCAGCGTCAGATGTGTATAAGAGACAGTTTTTAG-TTATGATGGGGGAGG	58	309
	R	GTCTCGTGGGCTCGGAGATGTGTATAAGAGACAG-CAAATCACACCTAAAACTCC		
BDNF_4 [a]	F	TCGTCGGCAGCGTCAGATGTGTATAAGAGACAGGAG-TTTATTTAGTATTTTGGATAGA	55.3	263
	R	GTCTCGTGGGCTCGGAGATGTGTATAAGAGA-CAGAAAAATCTATTCCAACCTACACC		
BDNF_5 [a]	F	TCGTCGGCAGCGTCAGATGTGTATAAGAGACAG-TTTTTTTAAGGGAAGGGGAGTT	54	216
	R	GTCTCGTGGGCTCGGAGATGTGTATAAGAGA-CAGAAACTAAAAATATTCTTCTCCACC		
BDNF_6 [a]	F	TCGTCGGCAGCGTCAGATGTGTATAAGAGACAGATAGAG-TTATTAATTAGTTGGA	55	295
	R	GTCTCGTGGGCTCGGAGATGTGTATAAGAGACAG-TAAATCCCTAAACTCCCTAAAA		
BDNF_7 [a]	F	TCGTCGGCAGCGTCAGATGTGTATAAGAGA-CAGGGTTTTAATGAGATATTTAT	58	220
	R	GTCTCGTGGGCTCGGAGATGTGTATAAGAGA-CAGAAAATCCCCCAATCAACTCTCT		

BDNF_8[a]	F	TCGTCGGCAGCGTCAGATGTGTATAAGAGACAGAAATGTT-GTTATTATTTTGATTGAATT	54	231
	R	GTCTCGTGGGCTCGGAGATGTGTATAAGAGA-CAGAACACCCAAATTCTCTAAAAAA		
BDNF_9[a]	F	TCGTCGGCAGCGTCAGATGTGTATAAGAGACAGNNTTTTTTA-GAGAATTTGGGTGT	56.7	279
	R	GTCTCGTGGGCTCGGAGATGTGTATAAGAGACAGNNAAAC-CTATCCTCACCTCCT		
COMT_1[a]	F	TCGTCGGCAGCGTCAGATGTGTATAAGAGA-CAGGTTTTTAATTTTGTATAGGTAAGAT	54	273
	R	GTCTCGTGGGCTCGGAGATGTGTATAAGAGACAG-TACCCTCCCTACCCACAAC		
COMT_2[a]	F	TCGTCGGCAGCGTCAGATGTGTATAAGAGACAGGGTTATTT-GTGGTTAGAAGTAGTT	54	308
	R	GTCTCGTGGGCTCGGAGATGTGTATAAGAGACAGAC-TACCCCAAAAACCCCAC		
COMT_3[a]	F	TCGTCGGCAGCGTCAGATGTGTATAAGAGACAGNNTGTAG-GAGGAGTATAGAGTATTGG	62	319
	R	GTCTCGTGGGCTCGGAGATGTGTATAAGAGA-CAGNNTCATAACCCACTCCTTCTACT		
SLC6A4_1	F	TCGTCGGCAGCGTCAGATGTGTATAAGAGACAGAGTTAAA-GAGTAGGAAAGTTAGGATTTT	54	298
	R	GTCTCGTGGGCTCGGAGATGTGTATAAGAGA-CAGACCCTCACATAATCTAATCTCTAAA		
SLC6A4_2	F	TCGTCGGCAGCGTCAGATGTGTATAAGAGACAGTTTGAGTAG-TTGGGAATATAAG	52	276
	R	GTCTCGTGGGCTCGGAGATGTGTATAAGAGA-CAGAAAACCCTTATCTAATTCCTCTC		

[a]Primer design according to Kouter et al. [56]

Table S3. Average DNA methylation values of amplicons; n/a: information not available.

BDNF1	*BDNF2*	*BDNF3*	*BDNF4*	*BDNF5*	*BDNF6*	*BDNF7*	*BDNF8*	*BDNF9*	*COMT1*	*COMT2*	*COMT3*	*SLC6A4_1*	*SLC6A4_2*
s01	3.04	1.87	3.45	10.70	7.09	6.63	1.96	1.54	1.13	97.19	0.59	75.61	0.96
s02	4.11	0.04	4.47	11.59	7.70	7.58	2.32	1.99	1.05	89.77	0.58	75.35	1.25
s03	4.23	0.30	9.20	13.55	7.90	9.63	3.07	2.22	1.74	92.77	0.66	75.91	1.25
s04	3.44	1.01	1.88	10.34	8.35	7.14	1.79	1.84	1.20	90.06	0.66	75.50	1.05

s05	3.53	0.03	4.33	12.81	9.27	8.72	2.42	2.93	1.34	89.48	0.48	73.34	n/a
s06	1.46	11.84	2.21	8.84	5.30	4.93	1.73	1.28	1.17	89.12	0.69	73.68	1.02
s07	3.96	1.46	3.49	11.64	6.36	6.29	1.58	1.00	1.10	n/a	0.77	73.86	1.18
s08	2.88	2.09	8.77	11.10	8.41	8.65	4.07	1.47	3.26	93.59	0.28	72.67	1.56
s09	2.71	n/a	1.32	11.03	6.58	n/a	1.69	0.99	1.07	92.51	0.54	73.18	0.93
s10	4.31	0.93	6.70	13.52	8.00	10.42	1.92	1.50	1.58	92.53	0.31	78.41	1.54
s11	2.49	0.07	4.15	12.22	8.91	7.66	1.14	2.25	1.33	89.45	0.46	75.16	1.36
s12	3.40	n/a	3.68	11.42	9.63	8.88	2.42	1.91	1.23	89.20	0.53	72.23	1.37
s13	0.84	2.35	2.03	9.27	5.83	5.00	2.06	1.62	0.79	96.15	0.32	76.98	1.26
s14	2.67	0.00	4.32	12.78	7.75	5.99	1.29	1.80	1.29	89.89	0.32	70.98	1.16
s15	5.04	n/a	5.51	13.76	9.15	12.92	2.87	1.98	1.53	89.30	0.47	72.02	1.74
s16	2.96	n/a	n/a	11.56	7.54	6.55	2.57	1.57	1.88	93.52	n/a	74.33	1.20
s17	4.37	14.56	8.44	13.25	11.60	10.21	2.52	2.90	2.05	93.88	0.97	66.86	1.43
s18	1.79	1.78	1.75	10.33	6.90	5.01	2.06	2.56	1.49	94.44	0.69	79.03	1.24
s19	1.61	0.04	2.84	11.57	6.43	5.16	1.97	1.29	1.70	95.91	0.97	78.54	1.33
s20	2.68	0.69	2.74	8.70	5.73	4.49	1.96	1.17	1.29	97.24	0.60	77.08	1.06
s21	3.71	0.56	3.60	11.08	7.73	7.43	3.42	2.23	1.66	90.41	1.04	73.51	1.55
s22	4.57	4.76	2.99	10.92	7.24	5.84	2.59	1.73	1.52	93.39	0.99	76.83	1.16
s23	2.84	8.34	3.20	10.73	7.25	6.27	2.73	2.25	1.58	93.29	0.92	75.19	1.58
s24	5.24	0.50	4.82	14.68	10.57	9.32	3.56	3.01	2.09	93.41	0.95	72.91	1.75
s25	n/a	1.78	4.11	10.83	7.16	5.39	2.82	1.98	1.86	93.70	1.06	78.47	1.32

Supplementary references

59. Kouter, K.; Nikolac Perkovic, M.; Nedic Erjavec, G.; Milos, T.; Tudor, L.; Uzun, S.; Mimica, N.; Pivac, N.; Videtic Paska, A. Difference in Methylation and Expression of Brain-Derived Neurotrophic Factor in Alzheimer's Disease and Mild Cognitive Impairment. Biomedicines 2023, 11, 235, doi:10.3390/biomedicines11020235.

Disclaimer/Publisher's Note: The statements, opinions and data contained in all publications are solely those of the individual author(s) and contributor(s) and not of MDPI and/or the editor(s). MDPI and/or the editor(s) disclaim responsibility for any injury to people or property resulting from any ideas, methods, instructions or products referred to in the content.
